# Comparison of Three Motor Subtype Classifications in *de novo* Parkinson's Disease Patients

**DOI:** 10.3389/fneur.2020.601225

**Published:** 2020-12-23

**Authors:** Jingru Ren, Ping Hua, Yuqian Li, Chenxi Pan, Lei Yan, Cuiyu Yu, Li Zhang, Pingyi Xu, Minming Zhang, Weiguo Liu

**Affiliations:** ^1^Department of Neurology, Affiliated Brain Hospital of Nanjing Medical University, Nanjing, China; ^2^Department of Geriatrics, Affiliated Brain Hospital of Nanjing Medical University, Nanjing, China; ^3^Department of Neurology, First Affiliated Hospital of Guangzhou Medical University, Guangzhou, China; ^4^Department of Radiology, Second Affiliated Hospital of Zhejiang University School of Medicine, Hangzhou, China

**Keywords:** *de novo* Parkinson's disease, motor subtype classifications, tremor-dominant, postural instability and gait difficulty, akinetic-rigid

## Abstract

**Objective:** The aims of this study were to compare the characteristics of three motor subtype classifications in patients with *de novo* Parkinson's disease (PD) and to find the most suitable motor subtype classification for identifying non-motor symptoms (NMSs).

**Methods:** According to previous studies, a total of 256 patients with *de novo* PD were classified using the tremor-dominant/mixed/akinetic-rigid (TD/mixed/AR), TD/indeterminate/postural instability and gait disturbance (PIGD), and predominantly TD/predominantly PIGD (p-TD/p-PIGD) classification systems.

**Results:** Among the TD/mixed/AR subgroups, the patients with the AR subtype obtained more severe motor scores than the patients with the TD subtype. Among the TD/indeterminate/PIGD subgroups and between the p-TD and p-PIGD subgroups, the patients with the PIGD/p-PIGD subtype obtained more severe scores related to activities of daily living (ADL), motor and non-motor symptoms, including depression, anxiety, and sleep impairment, than the patients with the TD/p-TD subtype. Furthermore, symptoms in the cardiovascular, gastrointestinal, and miscellaneous domains of the Non-motor Questionnaire (NMSQuest) were more prevalent in the patients with the PIGD/p-PIGD subtypes than the patients with the TD/p-TD subtypes.

**Conclusions:** The PIGD/p-PIGD subtypes had more severe ADL, motor and non-motor symptoms than the TD/p-TD subtypes. We disclosed for the first time that the TD/indeterminate/PIGD classification seems to be the most suitable classification among the three motor subtype classifications for identifying NMSs in PD.

## Introduction

Parkinson's disease (PD) is a highly clinically heterogeneous neurodegenerative disorder with wide variations in motor and non-motor manifestations (1). It is still unclear whether such heterogeneity merely reflects a diverse spectrum of clinical manifestations of a unitary disease or indicates the existence of disease phenotypes with distinctive clinical patterns and different pathophysiological abnormalities (2). Due to the lack of specific biomarkers in the diagnosis and progression of PD, an in-depth and comprehensive understanding of disease subtypes may be crucial to delineate disease mechanisms and ultimately improve tailored therapeutic strategies (3).

Among numerous empirical subtype systems based on clinical observation, including categories of age at onset, classifications of main motor phenotypes, and patterns of cognitive impairment and other non-motor symptoms (NMSs) (4), motor subtype classifications based on the prominent motor symptoms are the most commonly used (5). The clinical utility of three motor subtype classifications in PD, namely, tremor-dominant (TD)/mixed/akinetic-rigid (AR) (6, 7), TD/indeterminate/postural instability and gait difficulty (PIGD) (8, 9) and predominantly TD (p-TD)/predominantly PIGD (p-PIGD) subtype classifications (10, 11) has been extensively investigated. However, the terminology utilized to describe motor phenotypes of PD is equivocal and overlapping, and defining subtypes based on the ratio of two Unified Parkinson's Disease Rating Scale (UPDRS) subscores is arbitrary and intuitive, which contributes to an inaccurate body of published literature on the clinical roles of motor subtypes (12, 13). Therefore, the choice of motor subtype classifications is a key issue in the progression of clinical research on PD subtypes.

A recent study compared two motor subtype classifications of *de novo* PD patients and found that the TD/indeterminate/PIGD subtype classification appeared to be more likely to detect NMSs than the TD/mixed/AR phenotypes (14). Nevertheless, comparisons of three motor subtype classifications with *de novo* PD patients have not been examined previously. Furthermore, it is not known which of the three motor subtype classifications is the most suitable for identifying NMSs in patients with PD. Therefore, the purposes of this study were to compare demographic and clinical characteristics of the patients categorized using the three motor subtype classifications and to find the most suitable motor subtype classification for identifying NMSs.

## Methods

### Participants

A total of 256 newly-diagnosed untreated idiopathic PD patients from the Department of Neurology at the Affiliated Brain Hospital of Nanjing Medical University were recruited to participate in the study between January 2012 and June 2020. All participants were examined by a movement disorder specialist and diagnosed according to the United Kingdom Parkinson's Disease Society Brain Bank clinical diagnostic criteria (15). Patients showed positive responsiveness to levodopa treatment through the standardized acute levodopa challenge test and had at least one follow-up. Exclusion criteria comprised the following: (1) treated; (2) late-stage PD (modified Hoehn and Yahr (H-Y) stage >3); (3) atypical or secondary parkinsonism; (4) clinically significant lesions visible on brain magnetic resonance imaging (MRI) scans; (5) serious chronic diseases such as renal failure, heart failure, diabetes and its complications; and (6) difficulty in completing the clinical evaluation.

This study was approved by the Medical Ethics Committee of Affiliated Brain Hospital of Nanjing Medical University before study initiation and carried out in accordance with the Declaration of Helsinki. All individuals participating in the study provided written informed consent prior to participation in the experiment.

### Clinical Assessments

Demographic and clinical characteristics of PD patients were collected before starting dopaminergic treatment. Demographic characteristics specifically included age at assessment, sex, formal education, age at onset, and disease duration in years. The states of activities of daily living (ADL) were evaluated using part II subscales of the UPDRS. The UPDRS part III and modified H-Y stages were used to assess motor disability and disease severity, respectively. Global cognition was assessed with the Mini-Mental State Examination (MMSE) and Montreal Cognitive Assessment (MoCA). Mood was measured with the Hamilton Depression Scale (HAMD) and Hamilton Anxiety Scale (HAMA). Sleep was rated with the Parkinson Disease Sleep Scale (PDSS). NMSs were assessed by the Non-motor Questionnaire (NMSQuest) (16). The NMSQuest comprises 30 items, divided into nine domains, namely cardiovascular, sleep, mood/cognitive, perception/hallucinations, attention/memory, gastrointestinal, urinary, sexual function and miscellaneous (17).

### Motor Subtype Classifications

According to the three different methods described by Kang et al. (7), Stebbins et al. (18), and Herman et al. (10), the PD patients were categorized into the TD/mixed/AR, TD/indeterminate/PIGD and p-TD/p-PIGD classifications. In Kang's method, the ratio of the mean UPDRS tremor scores (UPDRS III items 20–21 divided by 4) to the mean UPDRS AR scores (UPDRS III items 22–27 and 31 divided by 15) was used to identify TD (ratio >1), mixed (0.8 ≤ ratios ≤ 1.0) and AR (ratio <0.8) PD patients (7). In Stebbins's method, the ratio of the mean UPDRS tremor scores (UPDRS II item 16 and UPDRS III items 20–21 divided by 8) to the mean UPDRS PIGD scores (UPDRS II items 13–15 and UPDRS III items 29–30 divided by 5) was used to identify TD (ratio ≥1.5), indeterminate (1.0 < ratios <1.5) and PIGD (ratio ≤ 1) PD patients (18). In Herman's method, a more stringent criteria was applied on the basis of the Stebbins method to identify the two representative subtypes with minimal symptom overlap, namely the p-TD and p-PIGD subtypes (10). If the PIGD score was higher than 3 or the tremor score was lower than 4, the patients were excluded from the TD group. Similarly, if the tremor score was higher than 3 or the PIGD score was lower than 4, the patients were excluded from the PIGD group.

### Statistical Analysis

Statistical analyses were performed using IBM SPSS software version 25.0. For demographic and clinical characteristics, the Kolmogorov-Smirnov test was used to check whether the data followed a normal distribution. The mean and standard deviation (SD) is presented for parametric variables, and the median and interquartile range (IQR) is presented for non-parametric variables. Categorical variables are presented as frequencies together with proportions. Among TD/mixed/AR and TD/indeterminate/PIGD subtypes, continuous data were compared using parametric (one-way analysis of variance, ANOVA) or non-parametric (Kruskal-Wallis *H*-test) tests, while categorical data were compared with Chi-square tests, followed by the Bonferroni correction for multiple comparisons. Between the p-TD and p-PIGD subtypes, continuous data were compared using parametric (two-sample *t*-test) or non-parametric (Mann-Whitney *U*-test) tests, while categorical data were compared with Chi-square tests. *P* < 0.05 was deemed to be significant.

## Results

The demographic and clinical characteristics of 256 *de novo* PD patients are summarized in Table 1. The PD patients had a mean age of 62.1 years old and a median PD duration of 2.0 years from the PD diagnosis.

**Table 1:** Demographic and clinical characteristics of *de novo* Parkinson's disease patients.

	**Overall (*n* = 256)**
Age (years)	62.1 ± 9.0
Sex (male)	131 (51.2)
Formal education (years)	9.0 (6.0, 12.0)
Age at onset (years)	59.4 ± 9.0
Disease duration (years)	2.0 (1.0, 3.0)
UPDRS ADL score	8.5 (5.0, 13.0)
UPDRS motor score	21.0 (14.0, 29.0)
H-Y stage	1.5 (1.0, 2.0)
MMSE	28.0 (26.0, 29.0)
MoCA	24.0 (20.0, 26.0)
HAMD	8.0 (4.0, 13.0)
HAMA	6.0 (3.0, 11.0)
PDSS	128.0 (110.0, 140.0)
NMSQuest	8.0 (5.0, 12.0)

*Data are given as mean ± SD, n (%) and median (interquartile range)*.

*UPDRS, unified Parkinson's disease rating scale; ADL, activities of daily living; H-Y, Hoehn and Yahr; MMSE, mini-mental state examination; MoCA, Montreal cognitive assessment; HAMD, Hamilton depression scale; HAMA, Hamilton anxiety scale; PDSS, Parkinson disease sleep scale; NMSQuest, non-motor questionnaire*.

Based on Kang's method of motor subtype classification, we identified 119 (46.5%) patients belonging to the TD subtype, 115 (44.9%) to the AR subtype, and only 22 (8.6%) to the mixed subtype. Among the TD/mixed/AR subgroups, there were no significant differences with respect to the demographic and clinical characteristics other than motor scores. In addition, in *post-hoc* analyses, the differences in the motor scores remained significant between the TD and AR subtypes, and the patients with the AR subtype obtained more severe motor scores than the patients with the TD subtype (Table 2).

**Table 2:** Comparison of the demographic and clinical characteristics of *de novo* Parkinson's disease patients among the tremor-dominant/mixed/akinetic-rigid subgroups.

	**TD (*n* = 119)**	**Mixed (*n* = 22)**	**AR (*n* = 115)**	***p*-value**	***post-hoc***
Age (years)	62.7 ± 9.4	61.4 ± 7.8	61.5 ± 8.7	0.540	
Sex (male)	65 (54.6)	13 (59.1)	53 (46.1)	0.315	
Formal education (years)	9.0 (6.0, 12.0)	9.0 (6.0, 15.0)	9.0 (6.0, 12.0)	0.984	
Age at onset (years)	59.7 ± 9.4	58.8 ± 8.1	59.1 ± 8.7	0.836	
Disease duration (years)	2.0 (1.0, 4.0)	2.0 (1.0, 3.3)	2.0 (1.0, 3.0)	0.365	
UPDRS ADL score	10.0 (5.0, 14.0)	7.0 (4.8, 11.8)	8.0 (5.0, 11.0)	0.196	
UPDRS motor score	20.0 (13.0, 28.0)	15.5 (13.8, 25.3)	23.0 (16.0, 32.0)	**0.014**	**0.038^a^**
H-Y stage	1.5 (1.0, 2.0)	1.5 (1.0, 2.0)	1.5 (1.0, 2.0)	0.466	
MMSE	28.0 (26.0, 29.0)	28.0 (26.0, 29.0)	28.0 (25.0, 29.0)	0.647	
MoCA	24.0 (20.0, 27.0)	24.0 (19.8, 26.0)	23.0 (19.0, 26.0)	0.169	
HAMD	8.0 (4.0, 14.0)	7.5 (3.8, 12.3)	9.0 (5.0, 15.0)	0.566	
HAMA	7.0 (3.0, 12.0)	4.0 (3.0, 12.0)	6.0 (3.0, 10.0)	0.728	
PDSS	123.0 (109.0, 139.0)	133.0 (101.5, 142.0)	130.0 (111.0, 140.0)	0.862	
NMSQuest	8.0 (5.0, 12.0)	7.5 (3.0, 10.0)	9.0 (5.0, 12.0)	0.388	

*Data are given as mean ± SD, n (%) and median (interquartile range)*.

*TD, tremor-dominant; AR, akinetic-rigid; UPDRS, unified Parkinson's disease rating scale; ADL, activities of daily living; H-Y, Hoehn and Yahr; MMSE, mini-mental state examination; MoCA, Montreal cognitive assessment; HAMD, Hamilton depression scale; HAMA, Hamilton anxiety scale; PDSS, Parkinson disease sleep scale; NMSQuest, non-motor questionnaire*.

*P-values calculated using ANOVA, Kruskal-Wallis H-test, or Chi-square test*.

*post-hoc calculated using the Bonferroni correction for multiple comparisons*.

a *Statistically significant between the tremor-dominant and akinetic-rigid subtypes*.

*Bold values are statistically significant (P < 0.05)*.

Based on Stebbins's method of motor subtype classification, we identified 140 (54.7%) patients belonging to the PIGD subtype, 78 (30.5%) to the TD subtype, and only 38 (14.8%) to the indeterminate subtype. Among the TD/indeterminate/PIGD subgroups, there were no significant differences with respect to the demographic or clinical characteristics, including age at assessment, sex, formal education, age at onset, disease duration in years, MMSE scores and MoCA scores. However, scores associated with ADL, motor and non-motor symptoms, including UPDRS ADL and motor scores, the modified H-Y stage, and HAMD, HAMA, PDSS, and NMSQuest scores significantly differed among the three groups. In addition, in *post-hoc* analyses, these differences remained significant between the TD and PIGD (or indeterminate) subtypes, and the patients with the PIGD subtype obtained more severe scores related to ADL, motor, and non-motor symptoms, including depression, anxiety, and sleep impairment, than patients with the TD subtype (Table 3).

**Table 3:** Comparison of the demographic and clinical characteristics of *de novo* Parkinson's disease patients among the tremor-dominant/indeterminate/postural instability and gait difficulty subgroups.

	**TD (*n* = 78)**	**Indeterminate (*n* = 38)**	**PIGD (*n* = 140)**	***p*-value**	***post-hoc***
Age (years)	61.1 ± 8.6	64.1 ± 9.4	62.1 ± 9.0	0.252	
Sex(male)	40 (51.3)	24 (63.2)	67 (47.9)	0.246	
Formal education (years)	9.0 (6.0, 12.0)	11.5 (6.0, 15.0)	9.0 (6.0, 12.0)	0.665	
Age at onset (years)	58.8 ± 8.8	60.8 ± 9.7	59.4 ± 8.9	0.530	
Disease duration (years)	2.0 (1.0, 3.3)	1.0 (1.0, 5.0)	2.0 (1.0, 3.0)	0.745	
UPDRS ADL score	7.0 (4.0, 11.0)	10.0 (6.0, 15.0)	9.0 (6.0, 13.0)	**0.005**	**0.011^a^, 0.029^b^**
UPDRS motor score	18.0 (11.0, 28.3)	24.5 (13.0, 31.5)	23.0 (15.3, 29.0)	**0.016**	**0.014^a^**
H-Y stage	1.0 (1.0, 2.0)	1.5 (1.0, 2.1)	1.5 (1.0, 2.0)	**0.018**	**0.045^a^, 0.047^b^**
MMSE	28.0 (26.0, 29.0)	29.0 (26.0, 29.3)	28.0 (25.0, 29.0)	0.530	
MoCA	23.5 (19.0, 26.3)	25.0 (21.0, 27.0)	23.0 (19.0, 26.0)	0.110	
HAMD	6.0 (2.0, 11.0)	8.0 (4.8, 13.5)	10.0 (5.0, 17.0)	**0.003**	**0.002^a^**
HAMA	5.0 (2.8, 9.3)	6.0 (3.0, 12.0)	7.5 (4.0, 11.0)	**0.047**	**0.041^a^**
PDSS	130.5 (118.8, 144.0)	127.5 (101.0, 143.3)	124.5 (100.5, 138.0)	**0.034**	**0.039^a^**
NMSQuest	7.0 (4.0, 10.0)	8.0 (4.8, 12.3)	9.0 (6.0, 12.0)	**0.006**	**0.004^a^**

*Data are given as mean ± SD, n (%) and median (interquartile range)*.

*TD, tremor-dominant; PIGD, postural instability and gait difficulty; UPDRS, unified Parkinson's disease rating scale; ADL, activities of daily living; H-Y, Hoehn and Yahr; MMSE, mini-mental state examination; MoCA, Montreal cognitive assessment; HAMD, Hamilton depression scale; HAMA, Hamilton anxiety scale; PDSS, Parkinson disease sleep scale; NMSQuest, non-motor questionnaire*.

*P-values calculated using ANOVA, Kruskal-Wallis H-test, or Chi-square test*.

*post-hoc calculated using the Bonferroni correction for multiple comparisons*.

a *Statistically significant between the tremor-dominant and postural instability and gait difficulty subtypes*.

b *Statistically significant between the tremor-dominant and indeterminate subtypes*.

*Bold values are statistically significant (P < 0.05)*.

Based on Herman's method of motor subtype classification, we identified 52 (20.3%) patients belonging to the p-TD subtype and 42 (16.4%) to the p-PIGD subtype. Between the p-TD and p-PIGD subtypes, there were no significant differences with respect to the demographic or clinical characteristics, including age at assessment, sex, formal education, age at onset, disease duration in years, MMSE scores and MoCA scores. However, scores associated with ADL, motor symptoms and non-motor symptoms, including UPDRS ADL and motor scores, the modified H-Y stage, and HAMD, HAMA, PDSS, and NMSQuest scores significantly differed between the two groups, and the patients with the p-PIGD subtype obtained more severe scores related to ADL, motor and non-motor symptoms, including depression, anxiety and sleep impairment, than patients with the p-TD subtype (Table 4).

**Table 4:** Comparison of the demographic and clinical characteristics of *de novo* Parkinson's disease patients between the predominantly tremor-dominant and predominantly postural instability and gait difficulty subgroups.

	**p-TD (*n* = 52)**	**p-PIGD (*n* = 42)**	***p*-value**
Age (years)	61.0 ± 9.5	61.0 ± 9.0	0.974
Sex (male)	24 (46.2)	18 (42.9)	0.749
Formal education (years)	9.0 (2.5, 12.0)	9.0 (6.0, 12.0)	0.673
Age at onset (years)	58.2 ± 9.7	58.1 ± 9.0	0.943
Disease duration (years)	2.5 (1.3, 4.0)	2.0 (1.0, 3.0)	0.259
UPDRS ADL score	7.5 (6.0, 11.8)	10.5 (8.0, 14.3)	**0.012**
UPDRS motor score	21.2 ± 9.4	26.7 ± 10.7	**0.018**
H-Y stage	1.5 (1.0, 2.0)	1.5 (1.5, 2.5)	**0.009**
MMSE	28.0 (26.0, 29.0)	28.0 (25.8, 29.0)	0.844
MoCA	23.0 (19.3, 26.5)	23.5 (19.0, 26.3)	0.798
HAMD	7.5 (4.3, 13.0)	10.0 (6.8, 19.8)	**0.019**
HAMA	5.5 (3.0, 10.8)	8.0 (5.0, 13.3)	**0.039**
PDSS	129.5 (116.0, 143.8)	123.0 (98.5, 136.3)	**0.039**
NMSQuest	7.0 (4.3, 10.0)	11.0 (5.8, 13.3)	**0.003**

*Data are given as mean ± SD, n (%), and median (interquartile range)*.

*p-TD, predominantly tremor-dominant; p-PIGD, predominantly postural instability and gait difficulty; UPDRS, unified Parkinson's disease rating scale; ADL, activities of daily living; H-Y, Hoehn and Yahr; MMSE, mini-mental state examination; MoCA, Montreal cognitive assessment; HAMD, Hamilton depression scale; HAMA, Hamilton anxiety scale; PDSS, Parkinson disease sleep scale; NMSQuest, non-motor questionnaire*.

*P-values calculated using two-sample t-test, Mann-Whitney U-test or Chi-square test*.

*Bold values are statistically significant (P < 0.05)*.

Since the NMSQuest scores were significantly different among the TD/indeterminate/PIGD groups and between the p-TD and p-PIGD groups, the nine domains of the NMSQuest were further compared across the two classifications. Differences in cardiovascular, gastrointestinal, and miscellaneous domains of the NMSQuest in the TD/indeterminate/PIGD classification were similar to the p-TD/p-PIGD classification, and the symptoms in these three domains were more prevalent in the patients with the PIGD/p-PIGD subtypes than the patients with the TD/p-TD subtypes (Table 5).

**Table 5:** Comparison of the non-motor symptoms in two motor subtype classifications of *de novo* Parkinson's disease patients.

	**TD (*n* = 78)**	**Indeterminate (*n* = 38)**	**PIGD (*n* = 140)**	***p*-value**	***post-hoc***	**p-TD (*n* = 52)**	**p-PIGD (*n* = 42)**	***p*-value**
Cardiovascular	22 (28.2)	18 (47.4)	67 (47.9)	**0.014**	**0.005^a^**	13 (25.0)	23 (54.8)	**0.003**
Sleep	58 (74.4)	29 (76.3)	121 (86.4)	0.064		41 (78.8)	38 (90.5)	0.126
Mood/cognitive	44 (56.4)	26 (68.4)	101 (72.1)	0.060		31 (59.6)	30 (71.4)	0.233
Perception/hallucinations	28 (35.9)	17 (44.7)	57 (40.7)	0.628		18 (34.6)	15 (35.7)	0.912
Attention/memory	58 (74.4)	30 (78.9)	105 (75.0)	0.854		42 (80.8)	30 (71.4)	0.288
Gastrointestinal	45 (57.7)	33 (86.8)	103 (73.6)	**0.003**	**0.016^a^, 0.002^b^**	30 (57.7)	37 (88.1)	**0.001**
Urinary	32 (41.0)	20 (52.6)	61 (43.6)	0.488		20 (38.5)	19 (45.2)	0.507
Sexual function	12 (15.4)	6 (15.8)	25 (17.9)	0.882		9 (17.3)	13 (31.0)	0.120
Miscellaneous	36 (46.2)	21 (55.3)	99 (70.7)	**0.001**	**<0.001^a^**	25 (48.1)	34 (81.0)	**0.001**

*Data are given as n (%)*.

*TD, tremor-dominant; PIGD, postural instability and gait difficulty; p-TD, predominantly tremor-dominant; p-PIGD, predominantly postural instability and gait difficulty*.

*P-values calculated using Chi-square test*.

*post-hoc calculated using the Bonferroni correction for multiple comparisons*.

a *Statistically significant between the tremor-dominant and postural instability and gait difficulty subtypes*.

b *Statistically significant between the tremor-dominant and indeterminate subtypes*.

*Bold values are statistically significant (P < 0.05)*.

## Discussion

To the best of our knowledge, this is the first paper exploring the distinction of the demographic and clinical characteristics of *de novo* PD patients among the three most commonly used motor subtype classifications. Among the three classification methods, patients with the non-tremor-dominant (AR or PIGD or p-PIGD) subtypes obtained more severe motor scores than the patients with the TD/p-TD subtypes. However, only patients with the PIGD/p-PIGD subtypes showed more severe NMSs than the patients with the TD/p-TD subtypes. Hence, compared with the other two classifications, the TD/mixed/AR subgroups may be unsuitable for identifying NMSs of PD patients, which is consistent with previous research results (14). Furthermore, differences in the NMSQuest domains between patients in the p-TD/p-PIGD classification were similar to those in the patients in the TD/indeterminate/PIGD classification. The p-TD/p-PIGD classification removes a large number of people, resulting in the loss of potentially significant information, but no more differences in non-motor features were found than with the TD/indeterminate/PIGD classification. Therefore, the TD/indeterminate/PIGD classification seems to be the most suitable classification among the three motor subtype classifications of PD for identifying NMSs.

The terms describing the PD motor subtypes overlap and are frequently puzzling. Jankovic et al. (8) first proposed that the PIGD subtype refers to a more representative subtype of axial symptoms presented in medical histories and observed in motor examinations compared with the TD subtype. Subsequently, Rajput et al. used AR terminology to represent a group of patients with PD whose axial and appendicular rigidity was more predominant relative to the TD subtype (19). Recently, Herman et al. used the p-PIGD/p-TD term to describe, with what appears to be the main goal, two more homogenous groups with minimal overlap of symptoms (20). In addition, the PD motor subtypes discussed above are based on clinical judgment and intuition, and the cutoffs used to define subtypes are arbitrarily selected (13). Although subtypes are determined according to the same principle by calculating the ratio between two UPDRS subscores, the UPDRS items and cutoffs for defining the subtypes are different in each study, so patients with PD may be categorized as a particular subtype according to one method and into another subtype with a different algorithm. Considering the vagueness of terminology and the arbitrariness of cutoffs, literature reports on the clinical role of PD motor subtype classification are inaccurate.

Clinically, however, almost all movement disorder experts still believe that these subtypes are credible, mainly because there is substantial evidence to support the link between these motor subtype classifications and relevant clinical features. In the TD/mixed/AR classification, patients with the AR phenotype were older at onset, had faster disease progression and had a higher cumulative incidence of cognitive impairment than patients with the TD subtype (19). In the TD/indeterminate/PIGD classification, patients categorized into the PIGD subtype had more severe motor symptoms, more NMSs including depression, fatigue, sleep impairment, urinary symptoms and sexual dysfunction, greater occupational disability, more aggressive disease progression and poorer prognoses than patients with the TD phenotype (8, 9, 21–23). In the p-TD/p-PIGD classification, patients with the p-PIGD subtype experienced more NMSs, greater autonomic function and poorer quality of life than patients with the p-TD subtype (11). Taken together, the PIGD/p-PIGD subtypes seemed to have more severe ADL, motor and non-motor symptoms than the TD/p-TD subtypes, which is consistent with our findings.

In this context, it is crucial to choose the appropriate motor subtype classification for further clinical research with PD patients. Our study compared the three motor subtype classifications of newly diagnosed PD patients, which eliminated the possible effects of drugs, and found that the TD/indeterminate/PIGD classification was the most suitable for PD clinical studies on NMSs. Because the presence of certain NMSs, such as hyposmia, constipation, depression and idiopathic REM sleep behavior disorder, has been recognized to significantly increase the risk of PD (24), the emerging concept of the prodromal phase of PD is based on not only motor symptoms but also NMSs (25, 26). Furthermore, there have been studies on the relationships between specific clusters of prodromal NMSs and the subsequent development of the PD motor phenotype (27). With the increasing clinical significance of NMSs, identifying subtypes of PD patients with unique motor and non-motor characteristics may provide a better understanding of the pathogenesis and help clinicians predict these symptoms and implement personalized, stratified treatment (28, 29).

In addition to motor subtypes empirically based on clinical observation, a data-driven approach with cluster analysis based on the co-occurrence of characteristics can also be used to define subtypes (30–32). Relative to motor subtypes, there are a priori hypotheses about how the characteristics of the disease are interrelated, but the data-driven approach does not involve any of these hypotheses, so it seems more unbiased. However, this novel approach still depends on certain choices, such as the variables selected to enter the analysis, the techniques of clustering, and the number of clusters found. Additionally, with these two methods, the exploratory results of motor subgrouping have not been consistent. Data-driven methods have provided support for the determination of TD subtypes, but they have failed to distinguish PIGD subtypes (33, 34). Therefore, when comparing subgroups of PD patients, clinicians should consider the characteristics of each classification.

When interpreting our results, several limitations need to be taken into consideration. First, most of the study participants were recruited from a single center in a clinical study, so it may not reflect the general population of PD and cannot be used for universalization. Nevertheless, the sample size of the study was sufficient for analysis. Second, the motor symptoms and non-motor abnormalities of *de novo* PD patients are generally mild, and a certain percentage of *de novo* PD patients show great variability in these motor subtype classifications (35, 36). Future studies can compare the clinical characteristics of different motor subtypes in the late stages of the disease. Third, the data we obtained are not comprehensive and may ignore potential factors that may have effect on ADL, motor and non-motor symptoms of PD patients, such as genetic and environmental attributes. Fourth, although *de novo* PD patients have at least one follow-up, they may still be mixed with patients with atypical parkinsonisms, so longer longitudinal follow-up is needed to distinguish atypical parkinsonisms from PD to improve the accuracy of PD diagnosis.

## Conclusion

In conclusion, the PIGD/p-PIGD subtypes had more severe ADL, motor and non-motor symptoms than the TD/p-TD subtypes. In addition, we demonstrated for the first time that the TD/indeterminate/PIGD classification seems to be the most suitable classification among the three motor subtype classifications of PD patients for identifying NMSs that is conducive to identifying subtypes with specific motor and non-motor symptoms relevant to the etiology, prognosis and response to subtype-specific treatment.

## Data Availability Statement

The original contributions presented in the study are included in the article/supplementary materials, further inquiries can be directed to the corresponding author.

## Ethics Statement

The studies involving human participants were reviewed and approved by Medical Ethics Committee of Affiliated Brain Hospital of Nanjing Medical University. The patients/participants provided their written informed consent to participate in this study.

## Author Contributions

WL organized the research project and critically revised for important academic content. JR collected data, designed, and performed statistical analysis, and drafted the preliminary manuscript. PH, YL, and CP helped in acquiring data. LY and CY critiqued the statistical analysis. LZ, PX, and MZ critically revised the manuscript. All authors contributed to the article and approved the submitted version.

## Conflict of Interest

The authors declare that the research was conducted in the absence of any commercial or financial relationships that could be construed as a potential conflict of interest.
